# Left bundle branch area pacing: anatomical and physiological features made easy

**DOI:** 10.1093/ehjopen/oeag081

**Published:** 2026-05-18

**Authors:** Guram Imnadze, Amr Abdin, Mauro Biffi, José-Ángel Cabrera, Óscar Cano, Jan De Pooter, Maxim Didenko, Marek Jastrzebski, Wilfried Mullens, Kevin Vernooy, Pugazhendhi Vijayaraman, Haran Burri

**Affiliations:** Department of Rhythmology, University Hospital Ruppin-Brandenburg, Brandenburg Medical School Theodor Fontane, Neuruppin 16816, Germany; Faculty of Health Science Brandenburg, Joint Faculty of Brandenburg, University of Technology Cottbus-Senftenberg, Brandenburg Medical School Theodor Fontane and University of Potsdam, Neuruppin, Rüdersdorf 16816, Germany; Clinic for Electrophysiology, Herz-Und Diabeteszentrum NRW, Ruhr-Universität Bochum, Bad Oeynhausen 32545, Germany; Cardiology, Angiology and Intensive Care Medicine, Internal Medicine Clinic III, Saarland University Hospital, Kirrberger Street 100, Homburg 66421, Germany; Cardiology Unit, Cardio-Thoracic and Vascular Department, S. Orsola University Hospital, University of Bologna, Bologna 40138, Italy; Departamento de Cardiología, Unidad de Arritmias, Hospital Universitario Quirón-Salud Madrid, 28223 Madrid, Spain; Departamento de Cardiologia, Hospital Universitario y Politecnico La Fe, Avinguda de Fernando Abril Martorell 106, Valencia 46026, Spain; Heart Centre, University Hospital Ghent, Corneel Heymanslaan 10, Gent 9000, Belgium; Clinic for Electrophysiology, Herz-Und Diabeteszentrum NRW, Ruhr-Universität Bochum, Bad Oeynhausen 32545, Germany; First Department of Cardiology and Electrocardiology and Arterial Hypertension, Jagiellonian University, College of Medicine, Kraków 31-008, Poland; Department of Cardiology, Ziekenhuis Oost-Limburg, Synaps Park 1, 3600 Genk, Belgium; Faculty of Medicine and Life Sciences, UHasselt, Biomedical Research Institute, LCRC, Diepenbeek 3500, Belgium; Department of Cardiology, Cardiovascular Research Institute Maastricht (CARIM), Maastricht University Medical Center, Universiteitssingel 50, Maastricht 6229, the Netherlands; Cardiology Department, Geisinger Wyoming Valley Medical Center, 1000 East Mountain Blvd., Wilkes-Barre, PA 18711, USA; Cardiology Department, Cardiac Pacing Unit, University Hospital of Geneva, Rue Gabrielle Perret Gentil 4, Geneva 14, CH-1211, Switzerland

**Keywords:** Left bundle branch area pacing, Conduction system pacing, Anatomy, Electrophysiology, Electrocardiography

## Abstract

Left bundle branch area pacing is being increasingly adopted in routine clinical practice as a more physiological alternative to right ventricular and biventricular pacing. Understanding the concepts of this pacing modality may be a hurdle for the non-electrophysiologist and specialists alike. This review article aims to explain in a didactic manner the anatomical and electrophysiological principles underlying left bundle branch area pacing.

## Graphical Abstract

**Graphical Abstract:**
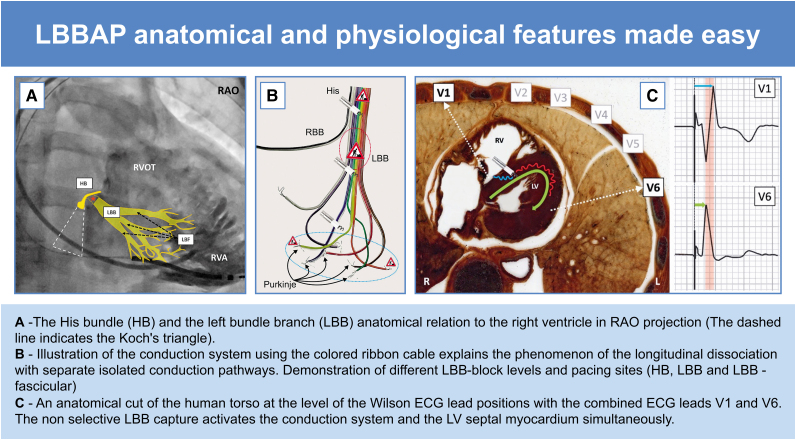

## Introduction

Pacing therapy for bradycardia, which was born in the second half of the last century, has evolved from pacing of the myocardium to a more physiological form which targets the intrinsic conduction system. The first experimental attempt at HBP was conducted in 1967,^1^ and it was applied permanently to humans in 2000.^2,3^ In 2021, it was incorporated into the ESC pacing guidelines.^4^ However, HBP has limitations, predominantly high pacing thresholds and sensing issues with a considerable proportion of patients require lead revision, even many years after implantation.^5–7^

In parallel to the development of HBP, the feasibility of permanent left ventricular septal pacing (LVSP) via a ventricular transseptal route was demonstrated in 2016 by Mafi-Rad *et al*.^8^ Shortly thereafter, Huang *et al*. described direct pacing of the proximal left bundle branch (LBB) using a similar transseptal approach as developed for LVSP.^9^ This marked a new era of physiological pacing with LBB area pacing (LBBAP), which is being increasingly adopted in routine clinical practice.^10–12^ The European Heart Rhythm Association (EHRA) has published a consensus document on CSP implantation technique,^13^ introduced this pacing modality in its core curriculum,^14^ and updated its indications in a recent consensus document.^15^ At present, LBBAP indications are spreading in both the pacing and cardiac resynchronization therapy fields.

As a consequence of conduction system pacing (CSP) adoption, there is increasing attention paid to the anatomy and physiology of the conduction system. The present article addresses the anatomical and electrophysiological aspects of the conduction system, to facilitate a more comprehensive understanding of LBBAP.

### Anatomy of the intraventricular septum and left bundle branch

Targeting the LBB from the right ventricular (RV) septum is more or less a blind process, and, as a consequence, knowledge of the correlative anatomy of the LBB and right-sided anatomical structures is crucial. The main anatomical landmarks of the right ventricular septum (RVS), which we can recognize during the procedure using fluoroscopy and electrograms (EGMs), are: The His bundle, the tricuspid annulus, RV apex, as well as its superior and inferior borders.

**Figure 1:**
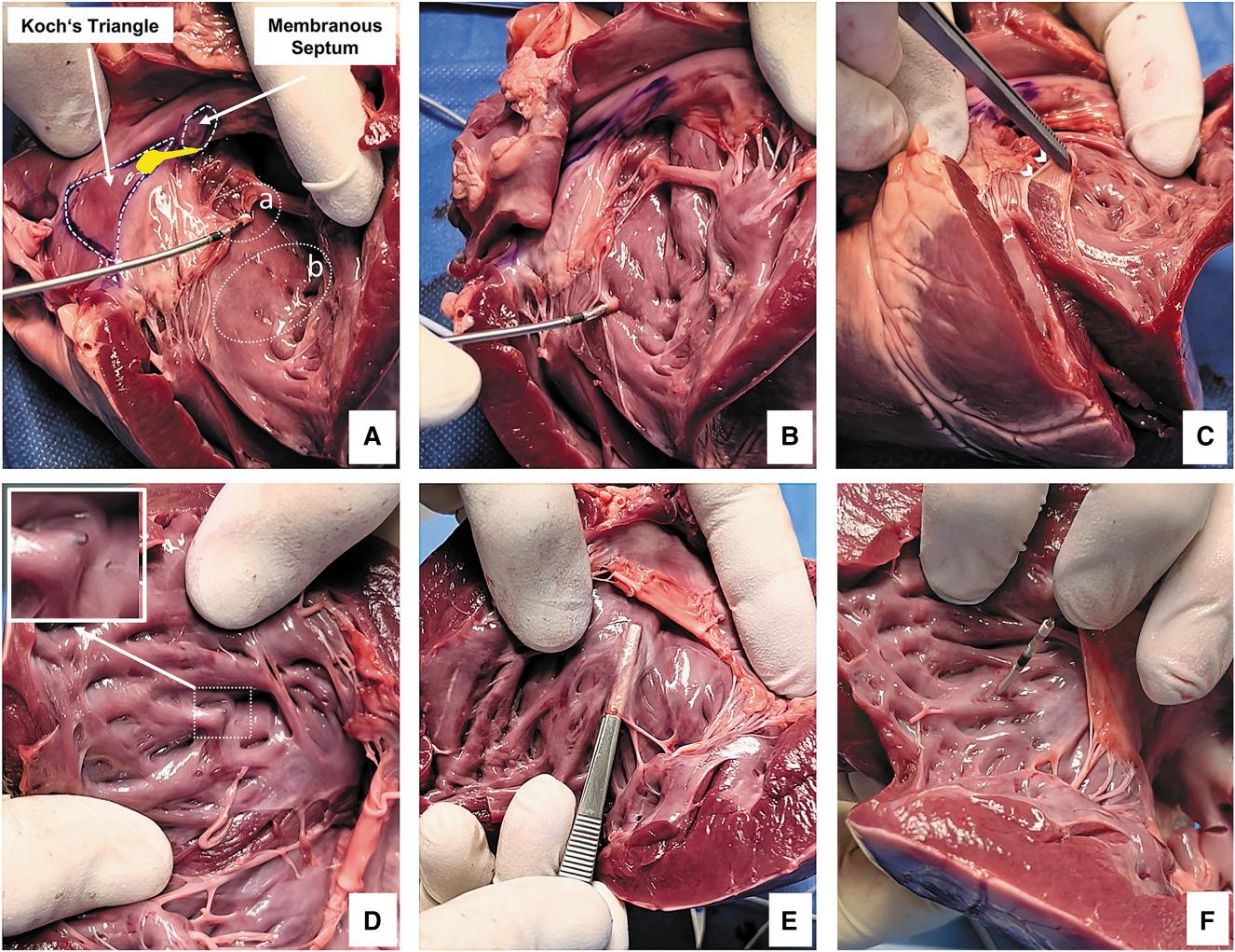
Demonstration of right and left interventricular septum with the porcine heart model. See also ***Video 1***. (*A*) *in situ* view of the RV and RA septum. The triangle of Koch and the membranous septum are indicated by the white dashed line. The AV node is depicted in yellow. **a** is the proximal insertion site for LBBP. Entrapment in a cordal structure is shown. **b** is the midseptal insertion site for LFP. The absence of cordal structures in this zone is clearly recognizable. (*B*) View of the RV septum, with entanglement effect of the lead. (*C*) the right ventricular endocardium is dissected from the myocardium. This thin but stiff layer is responsible for the entanglement and barrier effect. (*D*) The LV septum, with an optimal subendocardial position of the lead helix is shown. Despite pressure on the lead, perforation does not occur due to a barrier effect. (*E*) Separation of the stiff left ventricular endocardial layer. The Tweezers are now in the LV subendocardial space, where the conduction system lies. (*F*) Perforation of the lead into the LV cavity occurs only after excessive pressure and lead rotations. RV, right ventricle; RA, right atrium; AV, atrioventricular; LBBAP, left bundle branch area pacing; LBBP, left bundle branch pacing; LFP, left fascicular pacing.

**Figure 2:**
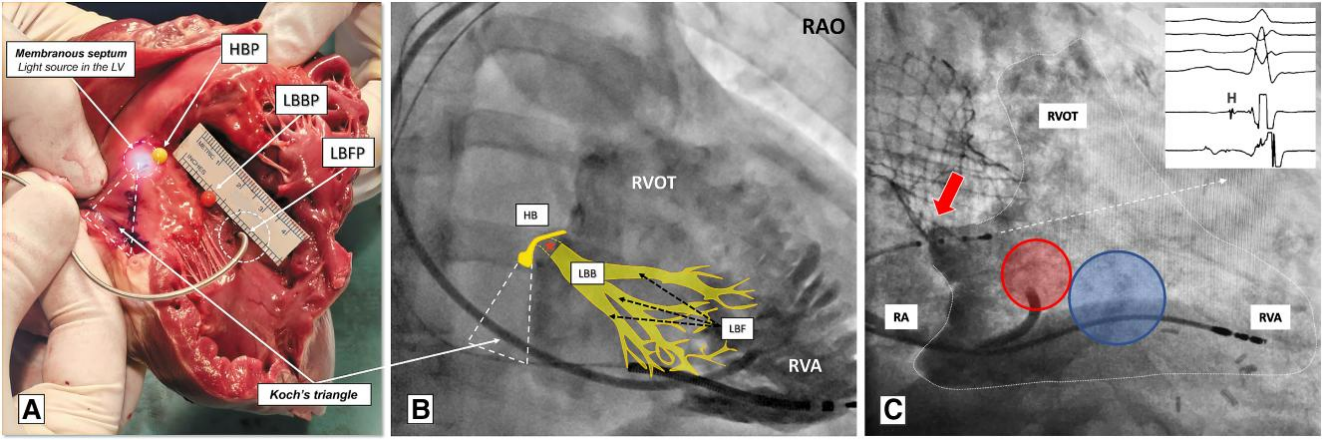
Macroscopic and X-ray anatomy correlation. (*A*) shows a porcine heart with a lateral wall incision of the right atrium and right ventricle, presenting the right atrial and ventricular septum from the right side. The dashed line delineates Koch’s triangle. The membranous septum is shown by transillumination from the left-sided subaortic position. The yellow pin indicates the position of the His bundle, and the red pin shows the basal insertion site for LBBAP. The lead is implanted into the middle septum, also known as the left bundle fascicular pacing side. (*B*) RAO X-ray projection of the patient during right ventricular angiography using the LBBAP sheath, which is in a slightly distal position. This view clearly demonstrates the entire right ventricular anatomy, including the trabeculation at the apical part, the right ventricle outflow tract (RVOT), and the tricuspid valve. The dashed line indicates the Koch’s triangle. The His bundle (bright yellow) runs anterior-superior, while the LBB (light yellow) tends to the ventricular apex after the bifurcation. (*C*) RAO X-ray projection of the patient during angiography of the right ventricle using the LBBAP sheath, in this case in a proximal position. This image offers a precise illustration of the tricuspid valve. The TAVI valve and diagnostic EP catheter indicate the His bundle position, which is in direct contact with the tricuspid valve annulus summit (red arrow). LBBAP can be achieved at the proximal (red circle) site of the RV septum (up to 20 mm from the His position towards the apex, targeting the main LBB) or at the middle part (blue circle) of the RV septum (30–40 mm from the His position towards the apex, targeting the fascicle level of the LBB).The *A* and *B* images were modified from Beyer SE *et al*. Inn Med 2024 with courtesy of Springer Medizin Verlag GmbH. LBB, Left bundle branch; LBF, left bundle fascicle; LBBAP, Left bundle branch area pacing; LBBFP, Left bundle fascicular pacing; HB, His bundle; HBP, His bundle pacing; RAO, right anterior oblique; RVOT, right ventricular outflow tract; RV, right ventricle; RVA, right ventricular apex; RA, right atrium; LV, left ventricle; EP, electrophysiology; TAVI, Transcatheter aortic valve implantation.

The anatomy of the RVS, predominantly in its proximal part, is complex and can lead to challenges during implantation. Anatomical structures such as the tricuspid valve leaflet, tendinous cords, papillary muscles, and endocardium, in some cases can wrap around the lead and result in entrapment, preventing deeper penetration into the myocardium. This can potentially result in damage to valvular structure or the lead itself (*Figures 1* and *2*, *Video 1*).

### LBB anatomy and physiology

The anatomy of the LBB has marked variability between individuals and depends on the relationship of the His bundle to the interventricular septum (IVS). The length of the membranous septum can also vary. The penetrating His bundle passes through the inferior margin of the membranous septum just after emerging from the compact AV node and marking the transition to the ventricular conduction system. This transition typically occurs between the right and non-coronary leaflets.^16–18^ The main LBB trunk then extends inferiorly 10 to 15 mm toward the apex. At the origin, the LBB can be broad or narrow (1–14 mm). Its thickness can be extremely variable from one to several cell layers of specialized smooth myocardial conducting tissue. On its way to the apex, the LBB widens, abruptly or more gradually. Mid and distal parts of the LBB contain more networked fibres than its proximal part.^18–21^ Histopathological investigations have confirmed the consistent presence of 3 rather than 2 main peripheral fascicles (*Figures 2* and *4*). The septal branch emerges in most cases from the common left bundle, but in some cases from the anterior or posterior fascicles. Some researchers in the past consider the LBB as an exclusively bifascicular structure with an anterior and a posterior ramification,^22^ with a network of fibres between the main trunk and both the anterior and posterior fascicles acting as the functional equivalent of a middle (septal) left fascicle.^23^ Based on this information, the main trunk of the LBB and its branching can be assumed to be unpredictable. When targeting the main LBB trunk, the lead should be inserted approximately up to 20 mm from the His bundle towards the apex. However, based on the variability of the LBB system anatomy and lead insertion site, the transseptal lead may often reach more distally, at the level of the fascicles, resulting in left fascicular pacing (LFP). This is the most common LBBAP capture type according to results from the MELOS study (about 70% of LBBAP cases).^24^

### The conduction system structure

**Figure 3:**
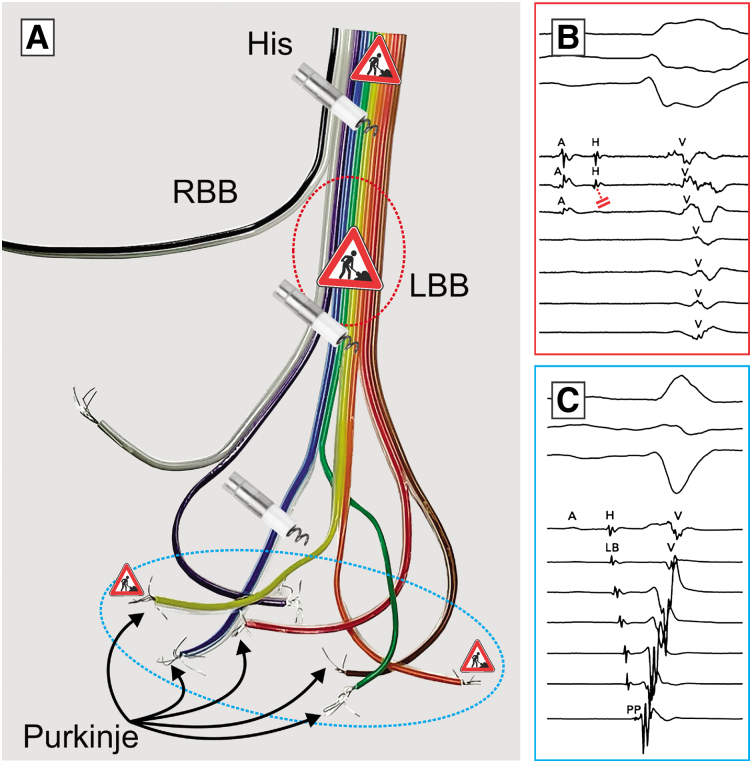
(*A*) Depiction of the conduction system using coloured cables. The traffic sign with construction works indicates a conduction block at different levels. The red dashed circle indicates the proximal left bundle branch site. The blue dashed circle indicates the mid-distal, fascicular level of the left bundle branch. The distal ends without isolation represent the Purkinje fibres. Once block occurs at the anatomical His level, but in predefined left bundle branch pathways, LBBB will occur. In such a scenario, stimulation of the His bundle distally to the block will effectively correct the block on the ECG. (*B*) The electrogram of LBB illustrates block at the anatomical LBB level (red dashed circle on figure *A*). (*C*) The electrogram of LBB illustrates LBB block-like ECG, without any blockade in the conduction system, but at the level of the LV endo-/myocardial tissue (blue dashed circle on the figure *A*). Panels *B* and *C* were modified from Tung R. *et al*. Arrhythm Electrophysiol Rev. 2020 with permission from Radcliffe Cardiology. RBB, right bundle branch; LBB, left bundle branch; PP, Purkinje Potential; H, his bundle potential; LB, left bundle potential; V, ventricular potential; A, atrial potential.

The His bundle and LBB are isolated from the myocardium and can be depicted as a ribbon, cable-like structure with several pathways which are isolated from each other,^25^ as shown in *Figure 3*. This helps understand the concept of longitudinal dissociation, whereby pathways at the His bundle level are already predestined for the right or left bundle branches and specific fascicles. In most cases of bundle branch block, the block occurs within the His bundle, and HBP distal to the block can correct the conduction disorder (detailed in *Figure 3*).

Insulation of the LBB network structures from the myocardium results in transmission of the action potential to the ventricular muscle at appropriate sites via the Purkinje fibres. This enables a correct activation sequence of the ventricular myocardium, which propagates rapidly.^25^ In contrast to the insulated proximal LBB, the very distal fascicular/Purkinje fibres are constituted by non-insulated structures, and thus capture of the distal conduction system might be easier. This is probably the explanation why it is possible to achieve conduction system capture even when a LBB/fascicular potential is not detected on the pacing lead EGM.^24^ It can also be explained by distant conduction system capture through a virtual electrode effect at a non-insulated level.^26^ Additionally, it has been proposed that due to its proximity, LFP can offer faster activation of the lateral LV wall than LBB pacing, resulting in shorter paced V6 R-wave peak time (RWPT) and shorter paced QRS duration.^25,27^ Moreover, at the fascicular level, direct septal depolarization can occur closer to the area of physiological activation of the septal myocardium, via the Purkinje system. The evaluation of an intramural gradient between the distal and the proximal parts of the LBB in the canine heart model during ventricular fibrillation showed that the distal conduction system, with a complex meshwork structure and non-insulated Purkinje fibres, presented shorter activation times in comparison to the proximal part.^28^

**Figure 4:**
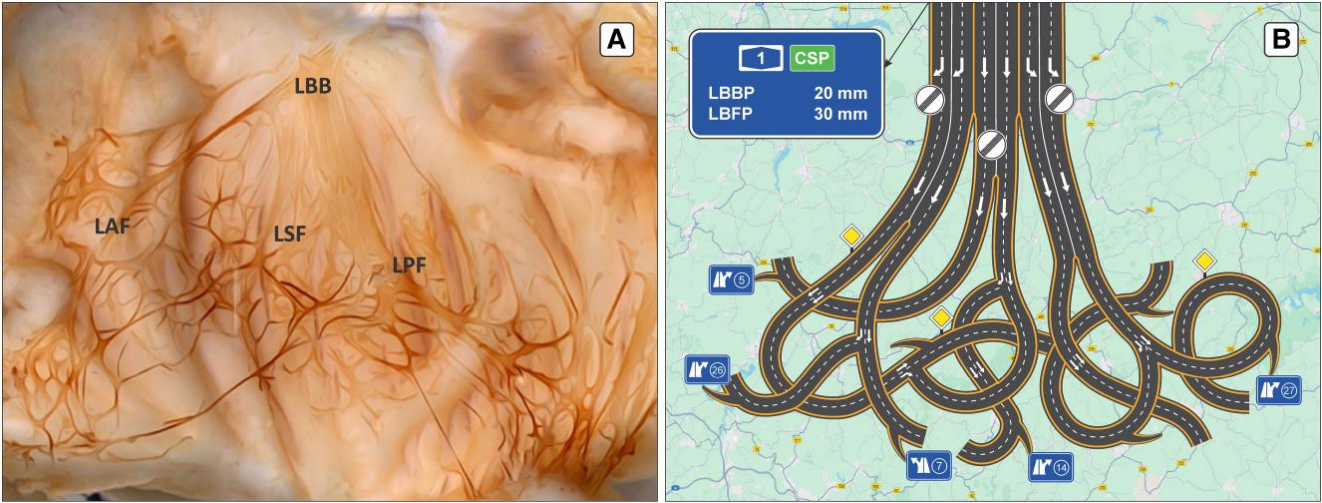
Anatomy of the left bundle branch, the highway model. (*A*) Macroscopic image of immunoenzyme-labelling (dark brown signal) of neurofilament (NF-M) on the endocardial surface of the left ventricle of the rabbit. (*B*) The highway model. The left bundle branch (LBB) and its fascicles are presented as a highway, while regular myocardium is shown as the country road. The highway exits represent the Purkinje cells. Panel *A* was modified from Atkinson A. *et al*. Moll Cell Cardiol. 2011 with the permission of Elsevier Ltd. LBB, left bundle branch; LAF, left anterior fascicle; LSF, left septal fascicle; LPF, left posterior fascicle; CSP, conduction system pacing; LBBP, left bundle branch pacing; LBFP, left bundle fascicular pacing.

To better understand the structure and function of the conduction system, we can also use the model of a highway as a comparison (*Figure 4*). The conduction system (His bundle, right and left bundle branches), which is electrically isolated from the myocardium, is very similar to a highway. Here, impulses are transmitted at high velocity. The myocardial tissue, on the other hand, can be thought of as a country road. The impulses, which travel simultaneously in all directions of the insulated conduction system, can only leave it via the Purkinje cells (similar to a highway exit) and thus excite the myocardium. The Purkinje cells are located in the mid-distal segments of both ventricles. Therefore, all ventricular walls are activated synchronously.

Additionally, a left bundle branch block (LBBB) pattern on the electrocardiogram (ECG) may not accurately reflect a block in the conduction system, but rather in the myocardium.^29^ In this case, LBB capture will not be effective to correct the conduction disorder.

### Layered structure of the interventricular septum and double endocardial barrier effect

The interventricular septum has multiple layers. While the myocardium constitutes the greatest part of the thickness of the septum, on each of the right and left ventricular surfaces there is a thin, semi-transparent, elastic and robust endocardial layer. In humans, the Purkinje system is placed subendocardially between the myocardium and the endocardial layers.^30,31^ To reach the LBB system, the lead must first penetrate the right ventricular septal endocardial layer, then traverse the entire myocardium, until the tip of the helix reaches the left-sided sub-endocardial region. However, care must be taken to avoid perforating the left-sided endocardium. These two endocardial layers might function as a barrier. Entanglement of the electrode within the RV endocardium can hinder septal penetration^13,32,33^ (*Figure 1*, *Videos 1* and *2*). Force is needed to penetrate this barrier, but once this is achieved, deeper advancement of the lead is much easier. As mentioned before, the LBB can have an extremely variable anatomy, but it always lies under the LV endocardial layer. The LV endocardium, characterized by its elasticity and robustness, likely exerts a beneficial effect by maintaining the position of the implanted lead and preventing its perforation into the LV cavity. This is supported by the observation of exceptionally low rates of late perforations following implantation.^24^

### Anatomical references for LBBAP

The first step during LBBP lead implantation is to determine the landing zone for the lead in the RV septum, facilitated by the identification of the His bundle position in the right anterior oblique (RAO 30°) projection using the pacing electrode itself. One can also use other anatomical landmarks as a reference. If the patient has a prosthetic aortic valve, then the lowest posterior part of the valve in the RAO 30° projection corresponds to the non-coronary sinus, and can be used as an indicator of the His bundle position. Another method to find the presumptive His bundle location is to perform angiography of the RV basal or middle segments by injecting contrast through the CSP delivery catheter to delineate the tricuspid valve and its annulus.^34^ The summit of the tricuspid valve annulus (TVA) in the 30° RAO projection also indicates the His bundle location. (*Figure 2*, *Video 3*).

### A comparison of electrocardiogram and electrogram findings with the corresponding anatomy

It seems logical to assume that if one obtains a right bundle branch block (RBBB)-like paced QRS morphology with an r/R’ in V1 and a relatively narrow QRS complex, LBB capture is obtained. The typical r’wave in V1 during LBBAP is just an expression of late RV activation, and not necessarily of LBB capture. Moreover, LBB capture without an r’wave is possible due to retrograde engagement of the RBB, or due to rapid transseptal conduction.

The most reliable confirmation of LBB system capture is the transition in QRS morphology with decrementing pacing output (see section below). which occurs in 30–90% of patients at implantation and more rarely at follow-up and correlates to the LBBP lead subendocardial position.^35,36^

**Figure 5:**
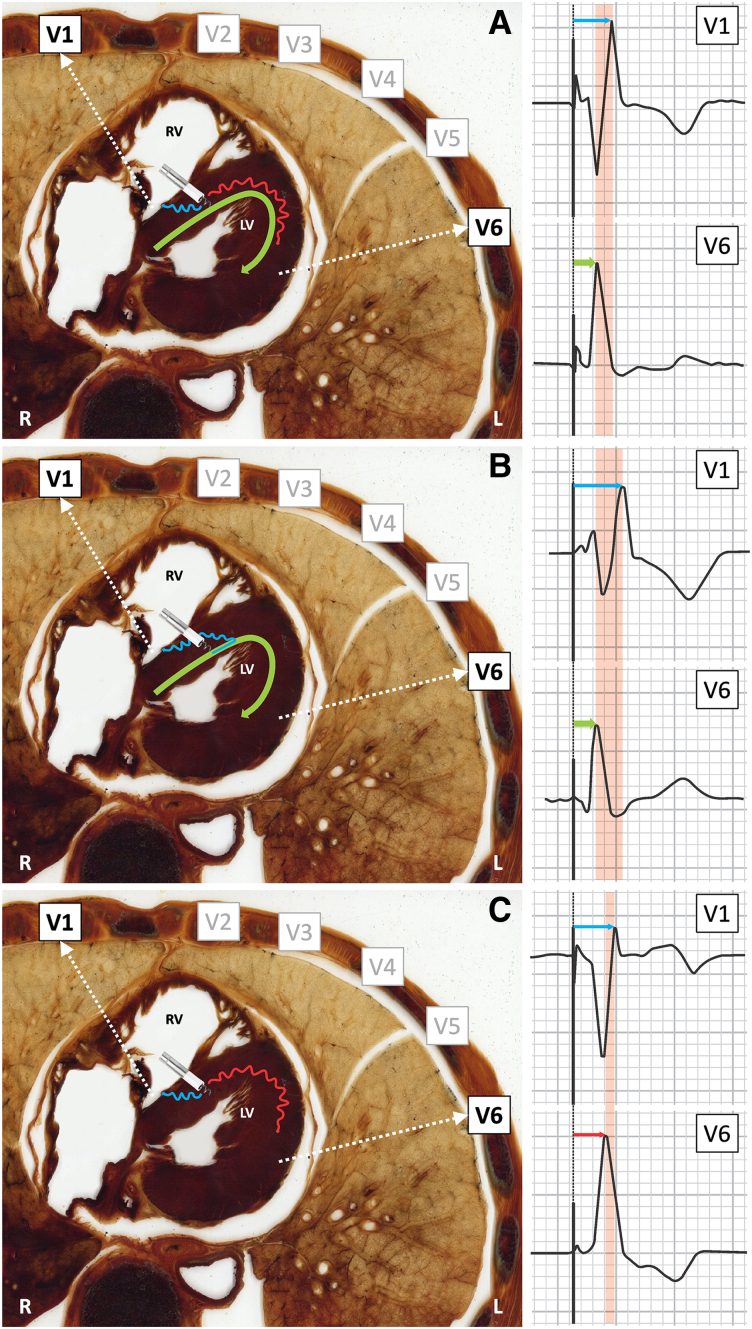
Correlation of cardiac anatomy and ECG for LBBAP. The left panel displays an anatomical cut (similar to a computer tomography view) of the human torso at the level of the Wilson ECG lead positions. On the right side, the combined ECG leads V1 and V6 demonstrate different types of capture. The stimulus captures the conduction system (bold green line) and/or myocardium (blue and red winding lines). The activation time is presented with corresponding coloured lines on the ECG. The V6-V1 interval is represented by pink colour. (*A*) ns-LBBP configuration, the conduction system and the LV septal myocardium are activated simultaneously. (*B*) s-LBBP—the capture occurs only in the conduction system. (*C*) LVSP scenario shows no capture of the conduction system, only the LV septal myocardium is activated. RV, right ventricle; LV, left ventricle; LBBP, left bundle branch pacing; Ns-LBBP, non-selective LBBP; s-LBBP, selective LBBP; LVSP, left ventricular septal pacing. The ECG images were modified from Jastrzebski M. *et al*. Europace 2021 with the courtesy of the Oxford University Press.

ECG criteria such as the delay between the pacing spike and the peak of the R-wave in V6 (V6RWPT), and the V6-V1 interpeak interval, are also used to evaluate LBB system capture. For better understanding it is helpful to superimpose the ECG vectors over the ventricular anatomy. As illustrated in *Figure 5*, V6RWPT indicates delay to activation of the lateral LV. If the V6RWPT is ≤75 ms, the activation of the lateral wall of the LV is likely due to activation of the conduction system, meaning that the activation occurs through the fast ‘highway’ rather than the slower regular myocardium. The validity and clinical relevance of this ECG criterion have been substantiated in recent publications and is currently employed in clinical practice.^37–39^

However, in patients with a cardiac resynchronization therapy indication, the conduction system and the left ventricle (LV) myocardial tissue may be diseased. As a result, the V6RWPT could be delayed, despite capture of the conduction system. In such cases, an additional ECG criterion, the V6-V1 interpeak interval, can overcome limitations of V6RWPT measurements.^40^ This is measured from the V6RWPT to the r/R’ -wave peak in lead V1 (V1RWPT), which indicates activation of the RV, used as a patient-specific reference point. If V6RWPT appears ≥44 ms earlier than V1RWPT, this indicates that fast LV activation is likely due to capture of the conduction system (*Figure 5*).

Another criterion for LBB capture in cases where an LBB/fascicular potential is recorded, is that the delay between the local potential and the V6RWPT is within 10 ms of that from the pacing spike to V6RWPT (i.e. the corresponding native and paced intervals are equal). The delay would be significantly longer if only cell-to-cell myocardial activation were to occur.

It is important to state that none of these criteria are 100% accurate, as they are subjected to variations in heart size, conduction velocity of the conduction system, and measurement error. For example V6RWPT and V6-V1 interpeak cutoffs have lower specificity for LBB capture diagnosis in patients with rS pattern in V6, because V6RWPT is misleadingly shortened and V1RWPT is lengthened by the relatively apical position of the pacing lead associated with such a QRS configuration (with the lead tip being closer to V6 and at greater distance from V1).

The gold standard criterion for conduction system capture is a transition in QRS morphology during decrementing unipolar pacing output, which leverages differences in capture thresholds between the conduction system tissue and the myocardium. These differences are more often seen during the first minutes after lead deployment. Another technique is to deliver programmed stimulation and leverage differences in refractory periods between the tissues, but this technique remains less popular as it requires some electrophysiological background and electrophysiology recording system availability.

Once the lead has been properly positioned in the left subendocardial space of the interventricular septum, three different variations of capture responses may occur and are described below.

### LBB capture patterns and transitions

Transitions with decrementing pacing output can be from ns-LBBP to s-LBBP (loss of myocardial capture) or from ns-LBBP to LVSP (loss of conduction system capture). As mentioned above, transitions confirming CSP capture can be seen in up to 90% of cases at implantation but are most often absent at follow-up at programmed pulse width (0.4 ms).^35,36^

**Non-selective LBBP (ns-LBBP**) indicates that, in addition to the conduction system, the LV septal myocardium is also captured. In case of conduction system capture, this activation pattern is almost always present at working pacing output (2.5 V/0.4 ms). (*Figure 5A*). The local myocardial activation exerts no influence on lateral LV activation (V6RWPT). This is due to the fact that this region is activated through the fast conduction system (highway). However, capture of the LV septal myocardium enables rapid activation of the RV septum due to their proximity, analogous to the short distance across a country road.

**Selective LBBP (s-LBBP)** signifies initial activation of the LBB/fascicle, without direct capture of the local myocardium (i.e. travel only via the highway). This is usually observed during implantation in the minutes following lead positioning and when the high current of injury of the local myocardium results in a higher stimulation threshold compared to that of the LBB. In *Figure 5A*, the ns-LBBP scenario is depicted. The stimulus captures the conduction system, causing activation of the LV lateral wall to occur rapidly (short V6RWPT). Although the RV is close to the stimulation site, activation of the LV lateral wall occurs >44 ms earlier than the RV free wall (long V6-V1 interpeak time). Once the output is decreased and the stimulation threshold of the local myocardium is reached, only the LBB is captured (see *Figure 5B*). In this scenario, the activation of the LV lateral wall occurs in a similar manner, unaltered short V6RWPT. The sole distinction is the RV activation which is delayed, with increased V6-V1 interpeak time. This is analogous to the situation encountered while driving on a highway and passing by one’s destination. In this scenario, immediate access to the destination (RV septum) is not possible. Instead, one must continue until the first exit (Purkinje fibre) is reached, at which point the journey resumes along the country road (myocardium) back to the destination. This results in a longer trip and a later arrival at the destination. Using the same logic, right ventricular activation is delayed and the r/R’ in V1 will appear later with s-LBBP, resulting in a longerV6-V1 interpeak interval as compared to ns-LBB. A transition in QRS morphology can be observed with decrementing pacing output with a delayed r/R’ in V1 without a significant change in the V6RWPT (*Figure 5B*). The EGM also shows splitting of the ventricular potential during transition to selective capture by virtue of delayed local myocardial depolarization at the lead tip.

**Left ventricular septal pacing (LVSP)** is basically a diagnosis by exclusion. If any LBB capture criteria are missing but the lead has reached the LV subendocardium with only myocardial capture, this can be considered as LVSP (*Figure 5C*). ECG criteria for conduction system capture are absent, however with presence of a terminal r/R’ indicating delayed right ventricular activation due to capture of the left ventricular septum.

Conversely, if LBB capture is lost before local myocardial LV septal capture, activation of the LV lateral wall will be delayed (longer V6RWPT). This is because the impulse will not take the fast conduction system, but rather the slower myocardium (*Figure 5C*). Concurrently, activation of the septum and RV remain unchanged, leading to a significant shortening of the V6-V1 interpeak interval with transition from ns-LBBP to LVSP.

In summary, during decremental unipolar output pacing, two possible QRS transitions can be identified: ns-LBBP to s-LBBP, and ns-LBBP to LVSP. Any of these two transition phenomena indicates that the conduction system was captured.

## Conclusion

CSP is more complex compared to standard pacing as it relies upon careful analysis of ECG and EGM signals and thus needs good understanding of conduction system anatomy and its electrophysiological characteristics. This modality is at the intersection of cardiac pacing and electrophysiology. A comprehensive understanding of the fundamental principles is instrumental in enabling physicians to enhance their knowledge and gain greater confidence in the interpretation of diagnostic findings. This, in turn, facilitates clinical decision-making processes.

## Data Availability

This material is available for open access.
